# Development of predictive models for lymphedema by using blood tests and therapy data

**DOI:** 10.1038/s41598-023-46567-1

**Published:** 2023-11-13

**Authors:** Xuan-Tung Trinh, Pham Ngoc Chien, Nguyen-Van Long, Le Thi Van Anh, Nguyen Ngan Giang, Sun-Young Nam, Yujin Myung

**Affiliations:** 1 Department of Plastic and Reconstructive Surgery, Seoul National University Bundang Hospital, Seongnam, 13620 Republic of Korea; 2 Department of Medical Device Development, College of Medicine, Seoul National University, Seoul, 03080 Republic of Korea

**Keywords:** Computational biology and bioinformatics, Machine learning, Software, Diseases, Diagnosis

## Abstract

Lymphedema is a disease that refers to tissue swelling caused by an accumulation of protein-rich fluid that is usually drained through the lymphatic system. Detection of lymphedema is often based on expensive diagnoses such as bioimpedance spectroscopy, shear wave elastography, computed tomography, etc. In current machine learning models for lymphedema prediction, reliance on observable symptoms reported by patients introduces the possibility of errors in patient-input data. Moreover, these symptoms are often absent during the initial stages of lymphedema, creating challenges in its early detection. Identifying lymphedema before these observable symptoms manifest would greatly benefit patients by potentially minimizing the discomfort caused by these symptoms. In this study, we propose to use new data, such as complete blood count, serum, and therapy data, to develop predictive models for lymphedema. This approach aims to compensate for the limitations of using only observable symptoms data. We collected data from 2137 patients, including 356 patients with lymphedema and 1781 patients without lymphedema, with the lymphedema status of each patient confirmed by clinicians. The data for each patient included: (1) a complete blood count (CBC) test, (2) a serum test, and (3) therapy information. We used various machine learning algorithms (i.e. random forest, gradient boosting, decision tree, logistic regression, and artificial neural network) to develop predictive models on the training dataset (i.e. 80% of the data) and evaluated the models on the external validation dataset (i.e. 20% of the data). After selecting the best predictive models, we created a web application to aid medical doctors and clinicians in the rapid screening of lymphedema patients. A dataset of 2137 patients was assembled from Seoul National University Bundang Hospital. Predictive models based on the random forest algorithm exhibited satisfactory performance (balanced accuracy = 87.0 ± 0.7%, sensitivity = 84.3 ± 0.6%, specificity = 89.1 ± 1.5%, precision = 97.4 ± 0.7%, F1 score = 90.4 ± 0.4%, and AUC = 0.931 ± 0.007). We developed a web application to facilitate the swift screening of lymphedema among medical practitioners: https://snubhtxt.shinyapps.io/SNUBH_Lymphedema. Our study introduces a novel tool for the early detection of lymphedema and establishes the foundation for future investigations into predicting different stages of the condition.

## Introduction

Lymphedema refers to a group of pathologic disorders with the excessive accumulation of protein-rich fluid drained through the lymphatic system of the body^1,2^. These disorders arise from an imbalance between the lymphatic circulation's capacity and the demand for lymphatic flow. There are two types of lymphedema: primary (i.e. lymphedema due to congenital or inherited conditions) and secondary (i.e. lymphedema triggered by acquired damage that occurs after surgical lymph node dissection). Lymphedema negatively affects the quality of life of patients because it leads to adverse outcomes such as pain, arm/leg swelling, and arm/leg heaviness^3–5^. Early detection of lymphedema is crucial for effective disease management and the minimization of physical impairment and patient depression. Traditional methods for detecting lymphedema are limb circumference measurement, bioimpedance spectroscopy^6,7^, shear wave elastography^8^, and infrared perometry^9^. However, these methods often require substantial time and costs, particularly when diagnosing a large number of patients.

Machine learning-based detection of lymphedema currently assists doctors and patients in real-time monitoring of lymphedema^10–15^. Fu et al. proposed an artificial neural network model to predict lymphedema^11^. The model used 26 lymphedema symptom features to predict lymphedema in 355 American patients with an accuracy of 93.75%, sensitivity of 95.65%, and specificity of 91.03%. This model had detection accuracy that was significantly higher than bioimpedance spectroscopy^11^. Armer et al. similarly employed patient self-reports of lymphedema symptoms to predict breast cancer-related lymphedema using a logistic regression model^12^. However, reliance on patient self-reports may introduce errors, leading to data with reduced reliability. Bell et al. discovered that among 29,656 patient reports, 20% contained errors, with 40% of these errors being significant, particularly in relation to diagnoses, medical history, medications, physical examination, and test results^16^. These potential errors within patient-reported data could significantly compromise the accuracy of predictions. Wei et al. developed another predictive model for lymphedema based on a logistic regression algorithm^10^. This model used 24 lymphedema-associated symptoms to predict lymphedema in 533 Chinese patients with a sensitivity of 77.1%, specificity of 88.3%, and accuracy of 82.5%. In this study, Wei et al. provided an open-access web application for patients to real-time monitor their lymphedema status. Wang et al. developed a logistic-regression-based scoring system to predict arm lymphedema risk for 358 breast cancer patients using axillary lymph node dissection level, history of hypertension, surgery on dominant arm, radiotherapy, and surgical infection/seroma/early edema (sensitivity = 81.20%, specificity = 80.90%, AUC = 0.877)^13^. Penn et al. also used logistic regression to identify risk factors for lymphedema and found that number of lymph node metastases and circumferential difference were significant predictors for lymphedema (AUC = 0.920)^14^.

Current models used lymphedema-related symptoms developed and recognized by researchers^12,17^ to predict risk of lymphedema. The symptom features include swelling in the arm/hand/breast, heaviness, firmness, tightness, stiffness, pain/aching/soreness, numbness, tenderness, stiffness, redness, blistering, burning, stabbing, tingling, skin toughness or thickness, impaired mobility in shoulder/arm/elbow/wrist/fingers^11^. However, those observable symptoms, such as swelling and volume changes, are often absent in the initial stages of lymphedema^3,12,18^, posing obstacles in the early detection of lymphedema. Detecting lymphedema before these observable symptoms occur would be beneficial for patients, as it could potentially minimize the discomfort caused by these symptoms. For this purpose, relying solely on symptoms-based predictions may not be the most suitable approach. Regular blood tests and therapies such as radiotherapy and chemotherapy are commonly administered to breast cancer patients, and these data can be obtained before observable symptoms of lymphedema manifest. Compared to self-reported symptoms, data derived from these technical measurements in hospitals are likely to have lower error rates.

Motivated by early detection of lymphedema, this study aims to: (1) developing predictive models for early detection of lymphedema by using blood test and therapy data and, (2) providing medical doctors and patients an open-access web application for quick screening of lymphedema. For those purposes, we collected blood test and therapy data of patients and then developed predictive models by using commonly used machine learning algorithms (i.e. random forest, logistic regression, gradient boosting, decision tree, and neural network). By benchmarking predictive models from those algorithms, we selected the best performance model and implemented it into a web application for quick screening lymphedema.

## Materials and methods

### Study population

The approval of this study was obtained from the Institutional Review Board Statement of Bundang Seoul National University Hospital (approval number: B2007-624-101). We collected data from 2137 patients, including 356 patients having lymphedema and 1781 patients not having lymphedema.

### Data collection and cleaning

The lymphedema status of each patient was confirmed by clinicians and physicians using the medical records of patients. Data of each patient includes: (1) complete blood count (CBC) test, (2) serum test, and (3) therapy information. After cleaning the missing data, we obtained a data table of 28 parameters, including 16 CBC parameters (Table 1), three serum test parameters (Table 2), nine therapy parameters (Table 3), and one lymphedema status parameter.

**Table 1:** Summary of blood test data.

No	Variable	Unit	Full name	Control (n = 2246)	Lymphedema (n = 460)	p-value (*t* test)
1	MCH	pg	Mean corpuscular hemoglobin	29.86	29.44	0.001
2	MCV	fL	Mean corpuscular volume	90.43	89.51	0.001
3	Lymphocyte	Cells/mL	Lymphocyte	32.44	31.39	0.018
4	Hb	g/dL	Hemoglobin	13.05	12.89	0.021
5	MCHC	g/dL	Mean corpuscular hemoglobin concentration	32.99	32.86	0.039
6	Seg.neu	Cells/mL	Segmented neutrophil	58.79	59.83	0.040
7	Hct	%	Hematocrit	39.50	39.20	0.089
8	Monocyte	Cells/mL	Monocyte	6.42	6.58	0.103
9	Basophil	Cells/mL	Basophil	0.46	0.45	0.363
10	Eosinophil	Cells/mL	Eosinophil	1.80	1.75	0.516
11	MPV	fL	Mean platelet volume	10.16	10.19	0.532
12	RBC	Cells/mL	Red blood cell	4.38	4.39	0.564
13	ANC	Cells/mL	Absolute neutrophil count	3770.44	3795.98	0.744
14	WBC	Cells/mL	White blood cells	6.28	6.25	0.779
15	PCT	ng/mL	Procalcitonin	0.28	0.28	0.804
16	PLT	Cells/mL	Platelets	272.39	272.98	0.869

**Table 2:** Summary of serum data.

No	Variable	Unit	Full name	Control (n = 2246)	Lymphedema (n = 460)	p-value (*t* test)
1	Sodium	g/dL	Sodium serum	140.64	140.83	0.096
2	Chloride	g/dL	Chloride serum	104.24	104.11	0.255
3	Potassium	g/dL	Potassium serum	4.23	4.22	0.368

**Table 3:** Summary of therapy data.

No	Parameter Unit		Full name		Control (n = 2246)	Lymphedema (n = 460)	p-value (*t* test)
1	lnn		Number of lymph nodes harvested		8.27	18.58	< 0.001
2	Age		Age		55.90	55.83	0.899
3	fx		Radiation fraction		8.88	13.97	< 0.001
4	Gy		Amount of radiation (gray)		21.16	32.24	< 0.001
5	Sex		Gender	Female	n = 2241	n = 457	
				Male	n = 5	n = 3	
6	Recon		Breast reconstruction	No reconstruction	n = 1846	n = 416	
				TRAM flap	n = 171	n = 24	
				Implant	n = 229	n = 20	
7	Tax		Taxane-based chemotherapy	No taxane	n = 1124	n = 55	
				Type 1	n = 639	n = 179	
				Type 2	n = 483	n = 226	
8	che		Chemotherapy	No	n = 1311	n = 159	
				Yes	n = 935	n = 301	
9	axi		Axilla radiation therapy	No	n = 1820	n = 218	
				Yes	n = 426	n = 242	

### Models development and validation

Models were developed to predict the lymphedema status of patients (i.e. yes or no). Previous studies used logistic regression (LR) and neural network algorithms (NNET) for lymphedema prediction. In this study we also used those two and other commonly used algorithms such as random forest^19^ (RF), gradient boosting tree^20^ (XGB) and C5.0 decision tree (DT) for developing the predictive models. We used R version 4.2.0^21^ and Rstudio^22^ programs to analyze and develop predictive models of lymphedema. Installed and used R packages were: openxlsx^23^, svDialogs^24^, caret^25^, randomForest^26^, xgboost^27^, C50^28^, nnet^29^, shiny^30^.

We randomly split the clean dataset into training (80% data) and external validation sets (20% data). The splitting was repeated three times to obtain three random splits. In the training process, machine learning algorithms were applied to the training set via tenfold cross-validation^31^, in which the training data was randomly partitioned into 10 mutually exclusive subsets, with 9 subsets for training and one for internal validation. Different algorithms shared this tenfold cross-validation and used default settings without further tuning parameters. Because the data of lymphedema and non-lymphedema patients in this study is imbalanced (17% of lymphedema and 83% of non-lymphedema), we adjusted class weights and decision threshold to deal with imbalance problem. The class weight was based on ratio of lymphedema and non-lymphedema and added in the training process. Trained models provide probability (between 0.0 and 1.0) of a data being lymphedema or not. The decision threshold is the probability to decide a is lymphedema or not. It is set to 0.5 for balanced data, but in this study, it was adjusted to maximize balanced accuracy of trained models (between 0.17 and 0.30). After training and obtaining trained models, we applied them on the external validation set to validate the application of the trained models. Metrics for validating the performance of trained models were: balanced accuracy, sensitivity, specificity, precision, F1 score, and area under the curve (AUC) measured through the receiver operating characteristic (ROC) curve^32^.

### Web application for screening lymphedema

Based on the performance of developed models, we chose the best models for developing a web application. We developed the web application to assist medical doctors in screening lymphedema by using shiny package in R^30^. The address of the web application is: https://snubhtxt.shinyapps.io/SNUBH_Lymphedema. Source code of models and web application is available at: https://github.com/trinhxt/SNUBH_Lymphedema. Detail description of the web application is described in the “Result” section.

### Ethics statement

This study was approved by institutional review board of Seoul National University Bundang Hospital (IRB number B2007-624-101). All methods were carried out in accordance with the tenets set by the declaration of Helsinki. Informed consent was obtained from all patients and patient data was anonymized to protect confidentiality.

## Results

### Clinical and histopathological characteristics

A dataset of 2706 rows and 29 columns was obtained after data collection and data cleaning. Number of data rows (i.e. 2706) was higher than the number of patients (i.e. 2137) because some patients checked blood/serum/therapy diagnosis several times at the Seoul National University Bundang Hospital. Among 29 columns, 16 columns are CBC test variables (Table 1), three columns are serum test variables (Table 2), nine columns are therapy variables (Table 3), and one column was lymphedema status confirmed by medical records and clinicians. Most of the patients were female (99.6%). The average age of patients was 55.9 ± 11.4; the youngest patient was 27, and the oldest patient was 95 years old. Student *t* test was conducted to compare the mean difference between control and lymphedema groups. The p-values of the *t* test are shown in Tables 1, 2 and 3. Box plots and distributions of those comparisons were included in the Figs. S1–4. Nine numerical variables showing significant difference between control and lymphedema groups area: number of lymph nodes harvested (p-value < 0.001), amount of radiation (p-value < 0.001), radiation fraction (p-value < 0.001), mean corpuscular hemoglobin (p-value = 0.001), mean corpuscular volume (p-value = 0.002), mean corpuscular hemoglobin concentration (p-value = 0.041), hemoglobin (p-value = 0.021), segmented neutrophil (p-value = 0.031), lymphocyte (p-value = 0.018).

### Predictive models for lymphedema

**Figure 1:**
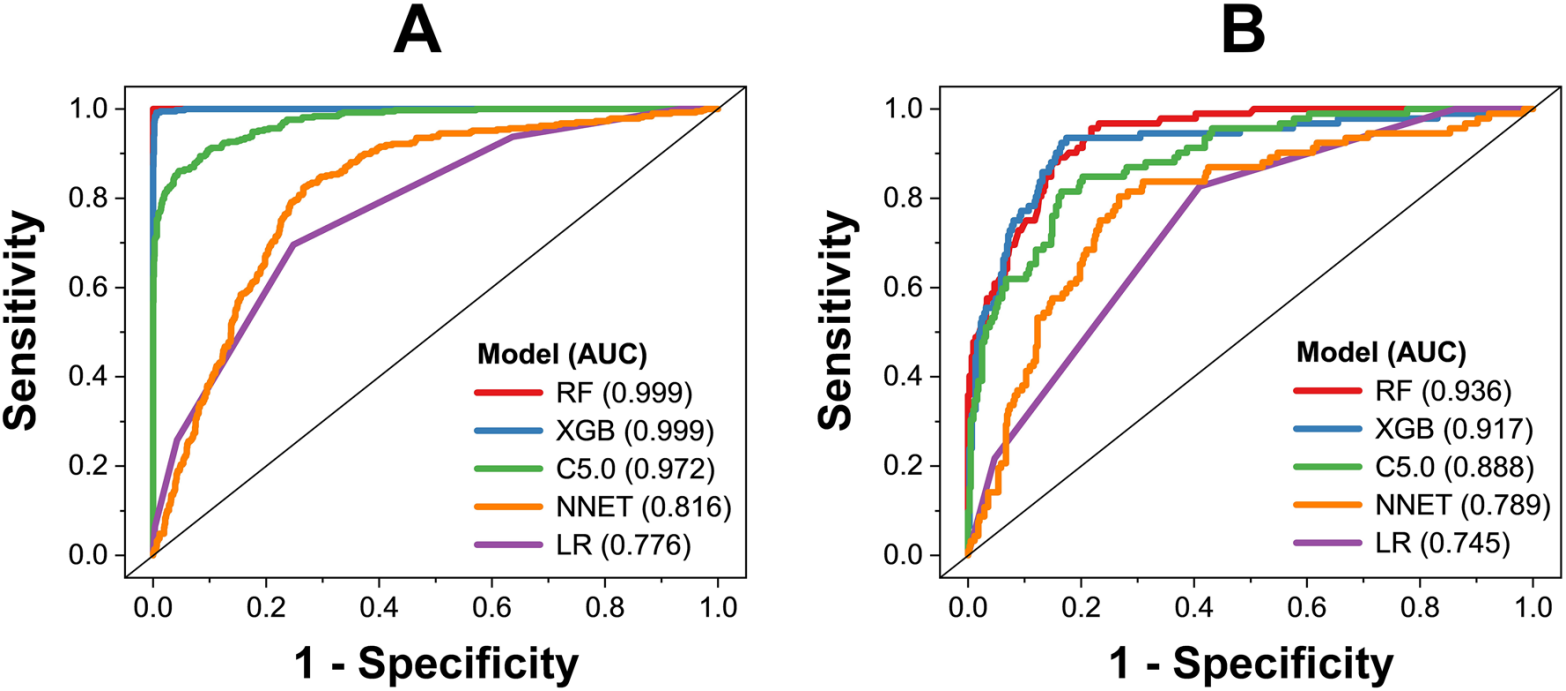
Receiver operating characteristic of predictive models on training dataset (**A**) and external validation dataset (**B**).

**Figure 2:**
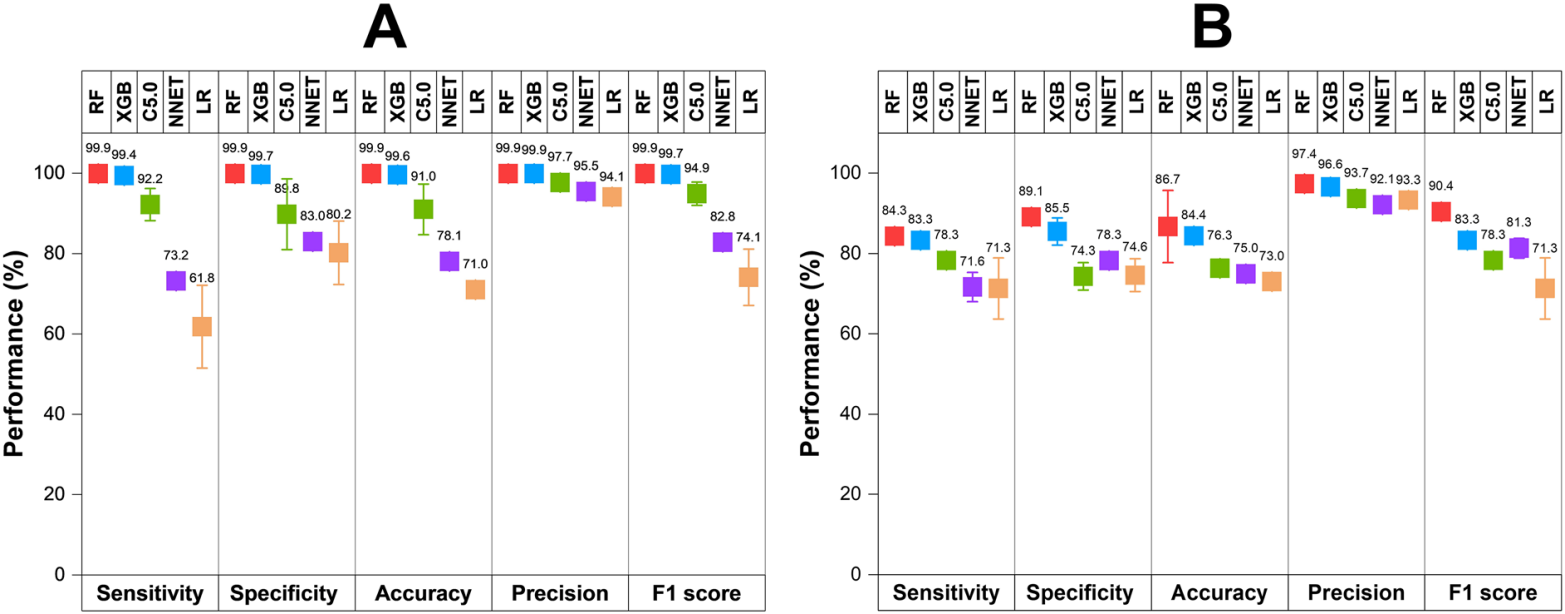
Performance metrics (sensitivity, specificity, accuracy, precision, and F1 score) of different models on the training dataset (**A**) and external validation dataset (**B**).

By using five algorithms (RF, XGB, C5.0, LR, and ANN), we obtained five predictive models. The performance of those models on the training and test datasets is shown in Figs. 1, 2. Among those models, RF model shows the best predictive performance on both training and external validation datasets, followed by XGB, C5.0, LR, and ANN models. For performance on the training dataset, the RF model shows that balanced accuracy = 99.9 ± 0.1%, sensitivity = 99.9 ± 0.1%, specificity = 99.9 ± 0.1%, precision = 99.9 ± 0.1%, F1 score = 99.9 ± 0.1%, and AUC = 0.999 ± 0.001. For performance on the external validation dataset, the RF model shows that balanced accuracy = 87.0 ± 0.7%, sensitivity = 84.3 ± 0.6%, specificity = 89.1 ± 1.5%, precision = 97.4 ± 0.7%, F1 score = 90.4 ± 0.4%, and AUC = 0.931 ± 0.007. Five algorithms were employed within the same framework of tenfold cross-validation, with no additional parameter tuning. Performance metrics were gathered from triplicate runs, revealing that the Random Forest (RF) model exhibited better performance, indicating that this was not by chance. Based on model performance, we chose RF model for further analysis of variable importance.

### Important variables to predict lymphedema

**Figure 3:**
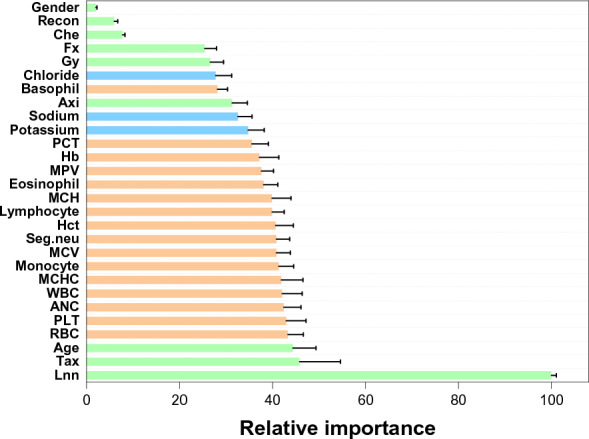
Relative importance of variables in deciding the risk of lymphedema for a patient. Results are based on the random forest model. Green variables are therapy, blue variables are serum, and orange variables are CBC parameters. Error bars are standard deviations of triplicates.

The relative importance of variables in deciding the risk of lymphedema for a patient was based on their variable importance in the RF model (calculated based on mean decrease accuracy method) and shown in Fig. 3. Among 28 variables of the RF models, the number of lymph nodes harvested (*Lnn*) is the most important, followed by taxane-based chemotherapy (*tax*), *age*, and other variables. The importance of the *Lnn* variable is almost as twice as the weight of *tax* and *age*, indicating that the *Lnn* variable is significantly important compared to other variables. High association between removal of lymph nodes and risk of lymphedema in this study agreed well with other cohort studies^16,33,34^. Removal of lymph nodes associated with risk of swelling and lead to risk of lymphedema^35^. Taxane-based chemotherapy is also confirmed to be associated with high risk of lymphedema^16^. The *t* test (Table 1, Fig. S1) indicated that some blood variable (mean corpuscular hemoglobin, mean corpuscular volume, lymphocyte, hemoglobin, mean corpuscular hemoglobin concentration, segmented neutrophil) showed significant difference between lymphedema and non-lymphedema with p-value < 0.05. However, these variables have low importance in the RF model because distribution of these variables did not show clear differences between lymphedema and non-lymphedema data (Fig. S2). Similarly, amount of radiation (Gy) and radiation fraction (fx) show low p-values (< 0.001, Fig. S3) but their distribution did not show clearly distinguish between lymphedema and non-lymphedema data (Fig. S4). Therefore, these data have low variable importance in RF models. In contrast, distribution of number of lymph nodes harvested shows clear differences between lymphedema and non-lymphedema data (Fig. S4).

### Applicability domain of predictive models

**Figure 4:**
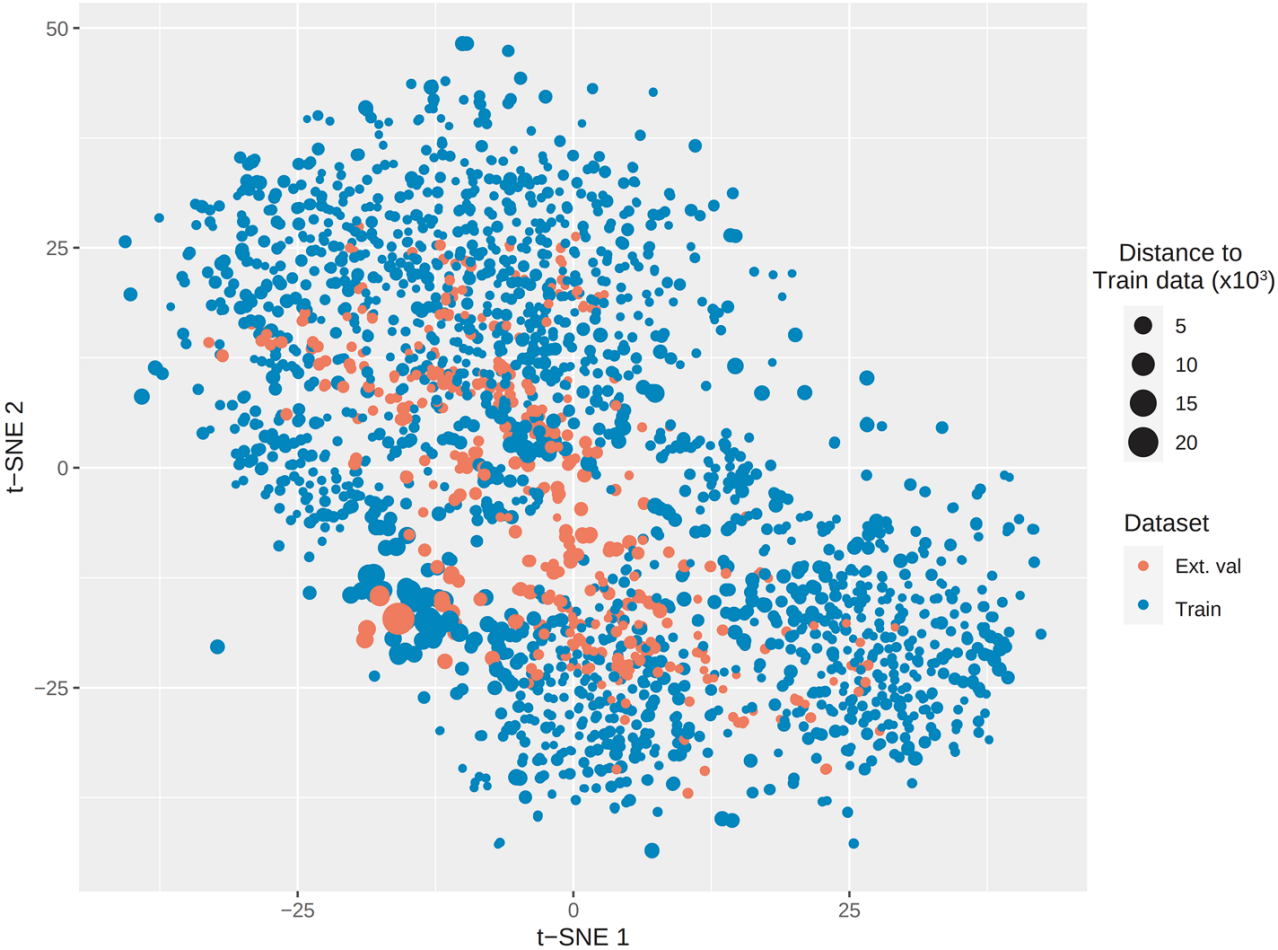
t-SNE visualization of training and test data. The size of the scatters is directly proportional to the Euclidean distance of data to the training dataset.

The applicability domain of predictive models is the region in the space of model variables (i.e. descriptors) representing the limitation of models toward new data^36,37^. In this study, by following other studies, we used the Euclidean distance method to define the applicability domain of our models^36–38^. Visualization of training and test datasets is shown in a t-SNE plot (Fig. 4) by using *snifter* package^39–41^. Data in the test set is within the applicability domain defined by the training dataset. If new data has a high Euclidean distance to the training set (over 2 × 10^4^), then the prediction on this data would have high uncertainty.

### Web application for screening lymphedema

**Figure 5:**
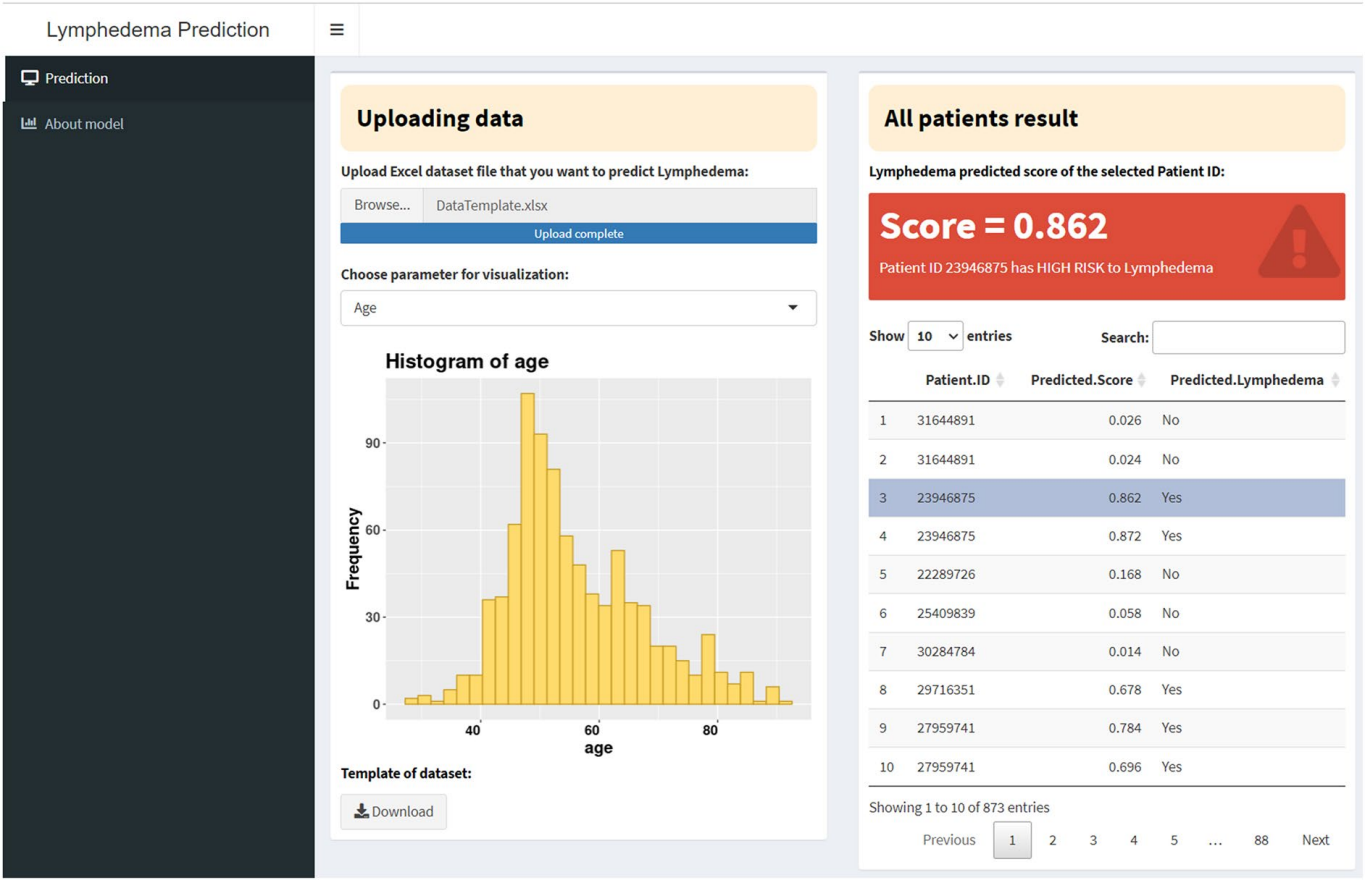
Web application for predicting risk to lymphedema of patients.

A web application, accessible at https://snubhtxt.shinyapps.io/SNUBH_Lymphedema, was developed using the shiny package in R. Users are required to upload a dataset to the platform, enabling the model to predict the risk of lymphedema for patients (Fig. 5). A dataset template is provided at the bottom left of the web interface (Fig. 5). Upon uploading the dataset, the model predicts a score for each patient based on their probability of developing lymphedema. A score (probability) exceeding 0.25 indicates a high risk of lymphedema for the patient, and vice versa. The decision to use 0.25 instead of 0.50 as the threshold is attributed to the dataset's imbalance, with 356 lymphedema patients out of a total of 2137 individuals. Setting the threshold at 0.25 ensures the models achieve the highest predictive performance. Users can select each patient to view their predicted score and receive suggestions regarding their risk of lymphedema.

## Discussion

Several studies have employed machine learning algorithms to develop models for predicting lymphedema^10–15^ (Table 4). They utilized machine learning algorithms such as logistic regression and artificial neural networks, using lymphedema symptoms to predict the onset of the disease. Our random forest model exhibits superior predictive performance in comparison to the models by Wei et al.^10^, Penn et al.^14^, and Wang et al.^13^ in terms of accuracy, sensitivity, specificity, and AUC. However, the model created by Fu et al.^11^ outperforms our model in terms of accuracy, sensitivity, and specificity. Notably, the dataset utilized in our study is considerably larger than other datasets, thereby providing models capable of predicting a broader range of patients. Consequently, our model holds significant potential for future predictive tasks.

**Table 4:** Comparison of models in this study and previous models.

	This study	Wei^10^	Penn^14^	Fu^11^	Wang^13^	Armer^12^
Algorithm	RF	LR	LR	ANN	LR	LR
Accuracy (%)	87.0 ± 0.7	82.5 ± NA	NA	93.8 ± 0.1	79.8 ± NA	NA
Sensitivity (%)	84.3 ± 0.6	77.1 ± NA	NA	95.7 ± 0.1	81.2 ± NA	NA
Specificity (%)	89.1 ± 1.5	88.3 ± NA	NA	91.0 ± 0.1	80.9 ± NA	NA
AUC	0.931 ± 0.007	0.889 ± 0.049	0.920 ± NA	NA	0.877 ± NA	NA
N_validation_	541	160	NA	71	NA	NA
N_train_	2165	373	342	284	358	80
No. variables	28	24	9	26	15	3
Type of variables	Blood, serum, therapy	Lymphedema symptoms	Surgery, therapy	Lymphedema symptoms	Surgery, therapy, BMI	Lymphedema symptoms
Data source	Clinical tests	Patient self-report	Clinical tests	Patient self-report	Clinical tests	Patient self-report
Lymphedema confirmation	Clinicians	Limb circumference	Limb circumference	Patient self-report	Limb circumference	Limb circumference
Web application	Yes	Yes	No	No	No	No

N_train_: number of patients in training dataset, N_validation_: number of patients in validation dataset.

Our model employed blood test and therapy data (e.g. the number of harvested lymph nodes, taxane-based chemotherapy) for predicting lymphedema. While the acquisition of these parameters might not be as immediate as patient self-report parameters used in other studies, the use of blood test and therapy data helps minimize human errors often associated with patient self-reports. Moreover, blood test and therapy data are more suitable for the early detection of lymphedema as they are not reliant on observable symptoms.

Given the differences in data collection, the model in our study could aid medical doctors and clinicians in the swift screening of lymphedema, while the other models might benefit patients by facilitating real-time self-monitoring of lymphedema. Future studies will aim to predict the stages of lymphedema, including early and late stages. Additionally, the confirmation of lymphedema in our study was conducted by clinicians and medical doctors based on the patients' medical records, rendering it more accurate than the patient self-reports and limb circumference measurements employed in other studies.

Determining the lymphedema status is a time-consuming task that requires clinicians and medical doctors to base their decisions on patients' medical records and costly diagnostic procedures, such as bioimpedance spectroscopy, shear wave elastography, computed tomography, and others. Rather than relying solely on these expensive techniques and meticulously reviewing the medical records of each patient, clinicians and medical doctors might utilize our models and web application for the rapid screening of patients with a potential high risk of lymphedema. Subsequently, the doctors can conduct a more thorough analysis of the medical records for those patients identified as having a high risk of lymphedema.

The dataset in this study encompasses 2137 patients, with 356 diagnosed with lymphedema and 1781 without the condition. We could expand this dataset further and update the predictive models in the future to ensure their applicability to a wider range of Korean patients. This study marks the initial step in the application of machine learning to the detection of lymphedema stages. According to the classification system of the International Society of Lymphology (ISL), lymphedema is categorized into four stages^5^. Stage 0 denotes a latent or subclinical condition where swelling is not apparent despite impaired lymph transport. Stage I signifies an early accumulation of fluid with relatively high protein content, which diminishes with limb elevation. Stage II indicates that limb elevation alone rarely reduces tissue swelling, with visible pitting. Stage III encompasses lymphostatic elephantiasis, where pitting is absent, and trophic skin changes such as acanthosis, fat deposits, and warty overgrowths develop. Classifying hundreds of lymphedema patients according to the ISL system would be a time-consuming task. Future studies will aim to develop predictive models for various stages of lymphedema.

## Conclusion

This study successfully developed machine learning models for predicting lymphedema using blood test and therapy data, making it more suitable for early detection of lymphedema in comparison to observable symptoms. The models, based on the random forest algorithm, exhibited satisfactory performance in predicting lymphedema. Our models utilized data collected from clinical tests, which were more reliable than patient-self-reported symptom data. We also developed an open-access web application to assist medical doctors in quickly screening for lymphedema. This study represents an initial step towards predicting the stages of lymphedema (i.e. stages I, II, III).

### Supplementary Information

Supplementary Figures.

## Data Availability

All datasets generated for this study are included in the article Supporting Information.

## Abbreviations

MCH: Mean corpuscular hemoglobin
MCV: Mean corpuscular volume
Hb: Hemoglobin
MCHC: Mean corpuscular hemoglobin concentration
Hct: Hematocriti
MPV: Mean platelet volume
RBC: Red blood cell
ANC: Absolute neutrophil count
WBC: White blood cells
PCT: Procalcitonin
PLT: Platelets
CBC: Complete blood count
