# Supplementary material for: Development of predictive models for lymphedema by using blood tests and therapy data
Source: Sci Rep. 2023 Nov 13;13:19720. doi: 10.1038/s41598-023-46567-1 (PMC10643602; doi:10.1038/s41598-023-46567-1)
Supplement: Supplementary file 1 — Supplementary Figures. [file 41598_2023_46567_MOESM1_ESM.docx]

**Development of predictive models for lymphedema by using blood tests and therapy data**

Xuan-Tung Trinh^1^, Pham Ngoc Chien^1^,Nguyen-Van Long^1^, Le Thi Van Anh^1^, Nguyen Ngan Giang^1,2^, Sun-Young Nam ^1*^, and Yujin Myung^1*^

^1^Department of Plastic and Reconstructive Surgery, Seoul National University Bundang Hospital. Seongnam 13620, Republic of Korea

^2^ Department of Medical Device Development, College of Medicine, Seoul National University, Seoul 03080, Republic of Korea

*Corresponding Author: [99261@snubh.org](mailto:99261@snubh.org); surgene@gmail.com

Supplementary material


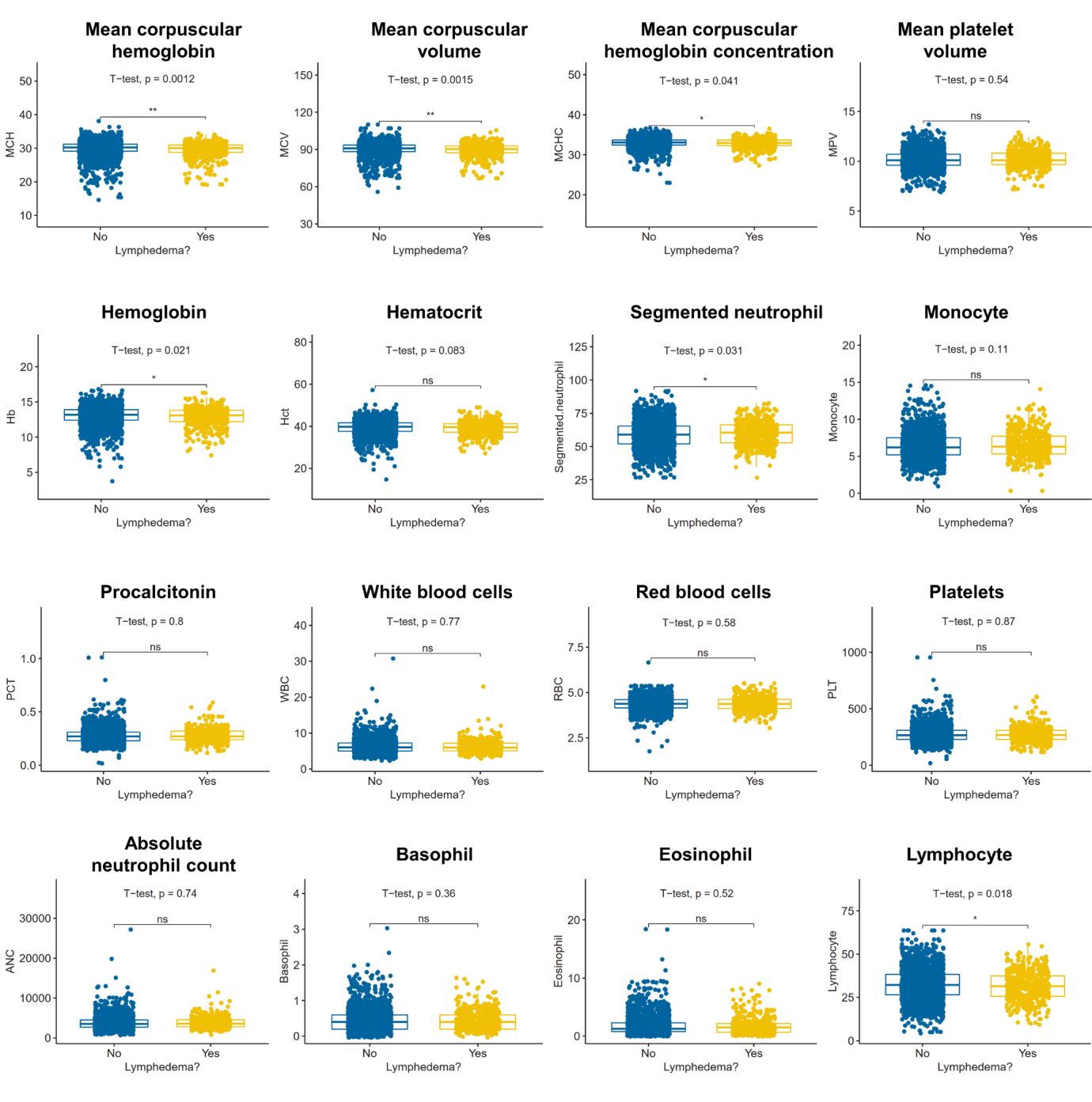


**Figure S1.** t-test for comparing control and lymphedema groups for CBC data.


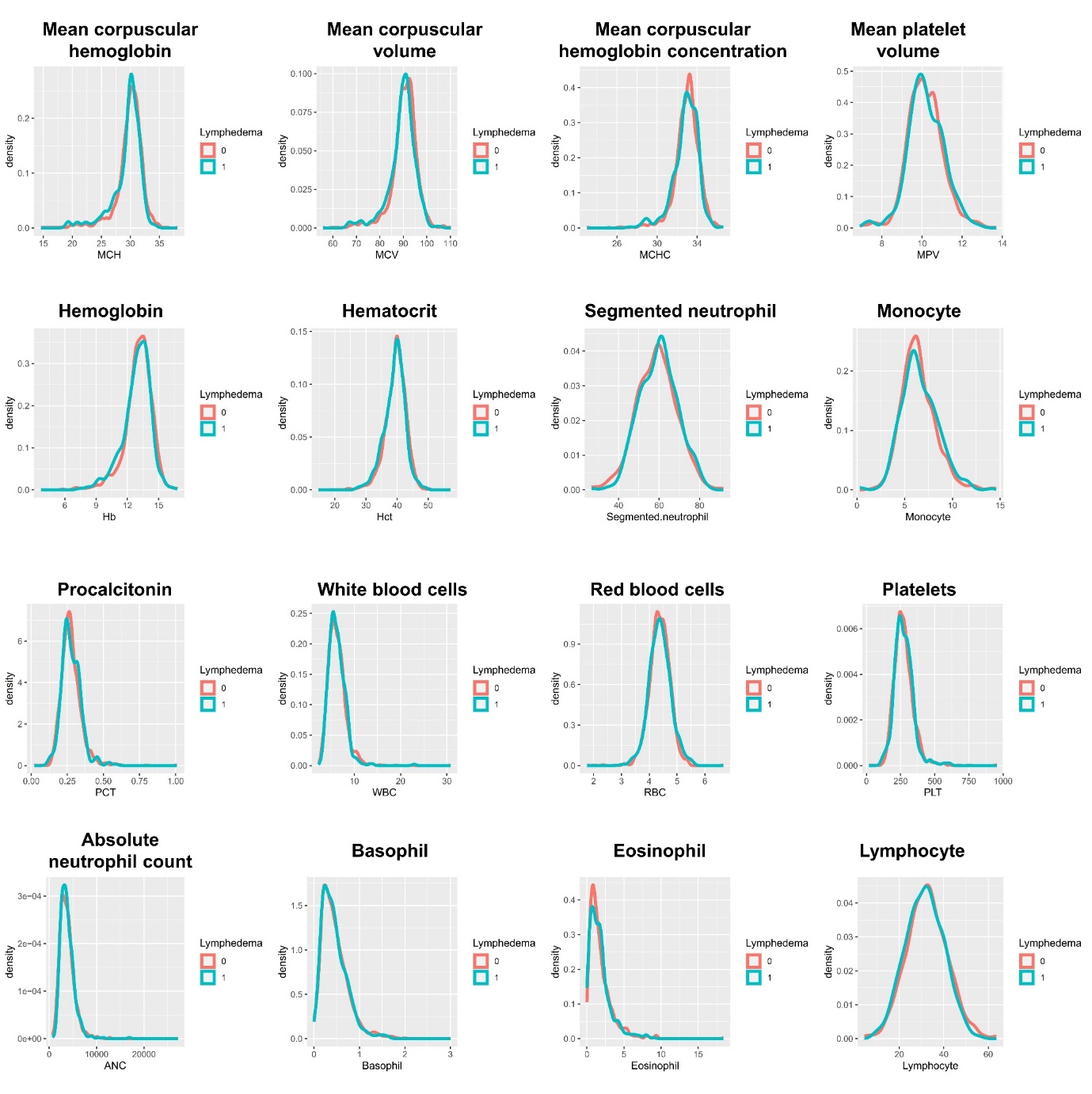


**Figure S2.** Distribution of control and lymphedema data regarding CBC data.


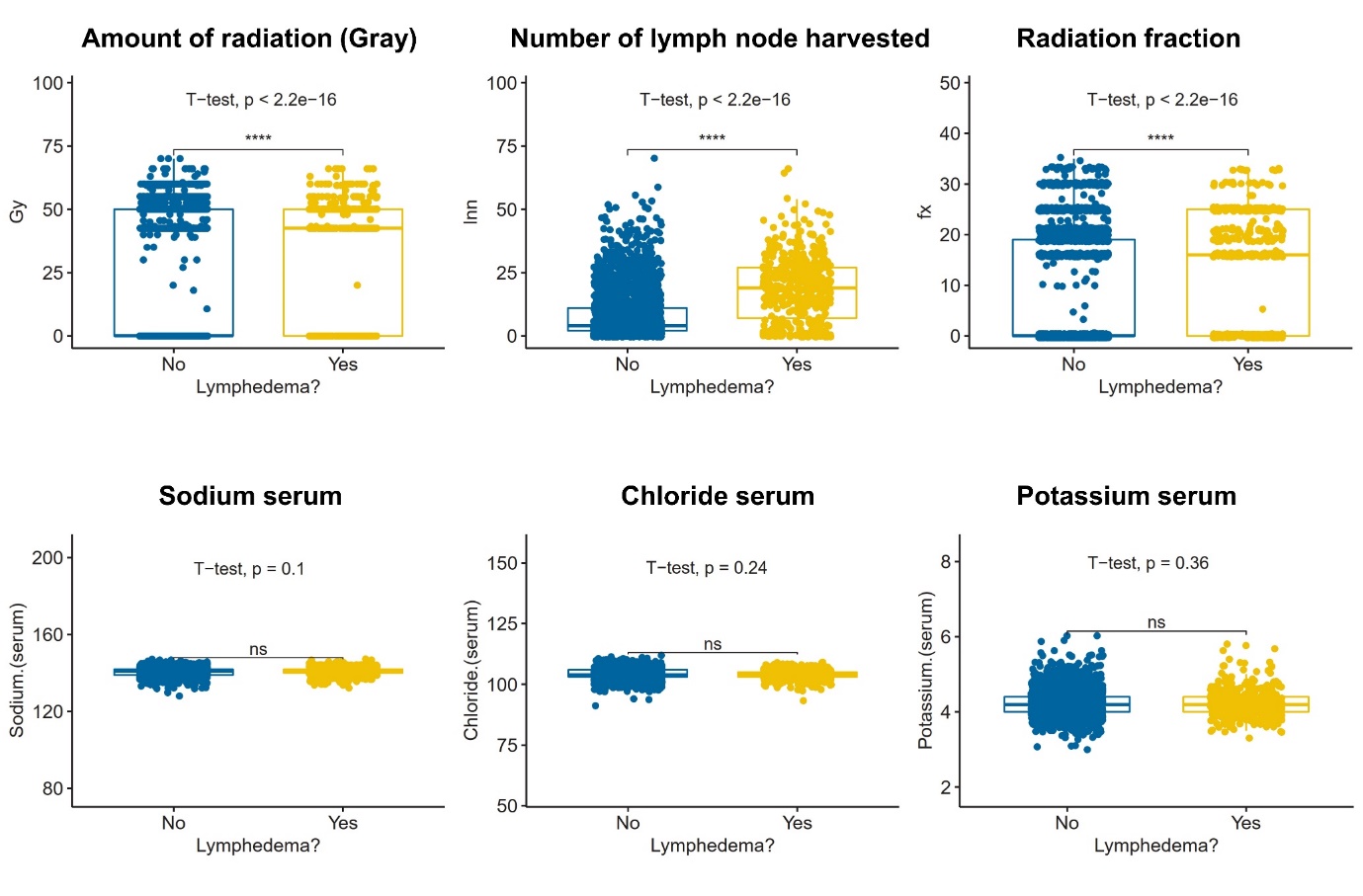
 **Figure S3.** t-test for comparing control and lymphedema groups for therapy and serum data


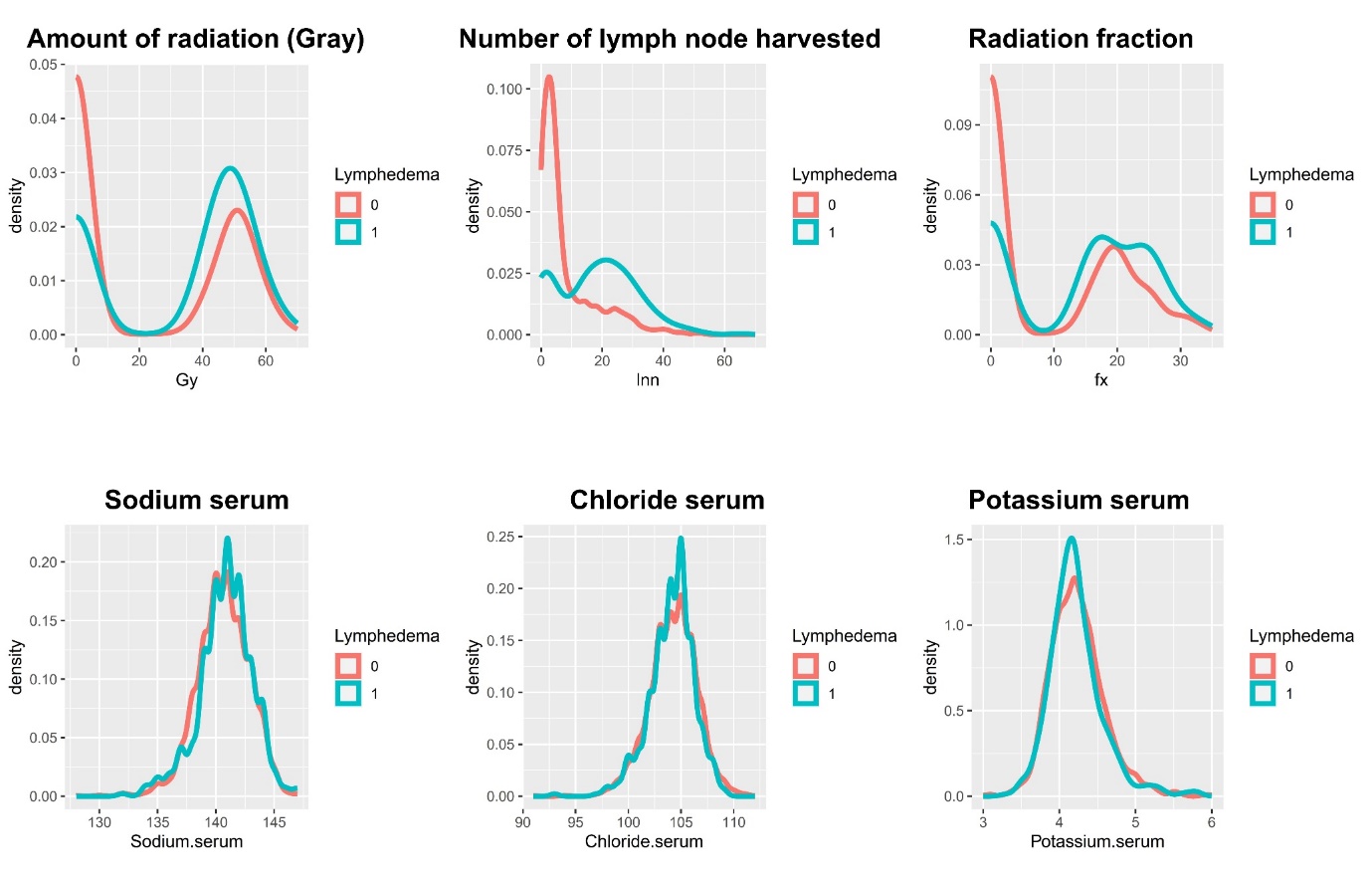


**Figure S4.** Distribution of control and lymphedema data regarding therapy and serum data
